# Data on nasal eosinophil positive rates in childhood asthma on each age

**DOI:** 10.1016/j.dib.2018.09.018

**Published:** 2018-09-13

**Authors:** Norihide Murayama, Kikuno Murayama

**Affiliations:** Murayama Paediatrics, 3-2-33 Nagayoshi-Nagahara-Higashi, Hirano-ku, Osaka-shi, 547-0013 Osaka, Japan

**Keywords:** Nasal eosinophils, Predictive factor for persistent asthma, Childhood asthma, Rhinitis, Bronchodilator

## Abstract

Nasal eosinophil examination is routine for the diagnosis of nasal eosinophil-positive rhinitis in patients with rhinorrhea symptoms. This retrospective data investigated whether testing for nasal discharge eosinophils is useful for diagnosing childhood allergic asthma, and changes of positive rates by each age.

Infants and young children (*n* = 180) with at least 3 recurrent episodes at intervals of ≥ 1 week of respiratory symptoms and bronchodilator inhalation improvements, were divided into an asthma group and a non-asthma group, and the presence or absence of nasal discharge eosinophils was examined by age. Correlations between nasal discharge eosinophils and other predictive factors for persistent asthma were also examined.

The evaluation of nasal discharge eosinophils in the asthma group showed a significantly high positive rate in older age groups than in the 0–1-year-old age group (*p*< 0.05–0.001). However, none of the asthma patient groups had any significant changes between the 0–1-year-old group and older groups. This pattern was similar for other risk factors, showing correlations between nasal discharge eosinophils and other predictive factors.

**Specifications table**

**Table:** 

Subject area	Clinical immunology
More specific subject area	Asthma in younger childhood
Type of data	Table and Figure
How data was acquired	Clinical examination
Data format	Analysed
Experimental factors	Asthma prognosis factor on each age
Experimental features	Nasal eosinophil positive rates in childhood asthma increased according to age raised.
Data source location	Osaka, Japan
Data accessibility	The data are supplied with this article
Related research article	Dissecting childhood asthma with nasal transcriptomics distinguishes subphenotypes of disease. Poole A et al. J Allergy Clin Immunol. (2014).
	Childhood asthma control test (C-ACT) and nasal eosinophil inflammation in asthmatic children. Piacentini GL et al. Allergy. (2010)
	Virus detection and cytokine profile in relation to age among acute exacerbations of childhood asthma. Kato M et al. Allergol Int. (2015)

**Value of the data**

• Nasal eosinophil positive rates with asthma symptoms increased by age raised.
• Nasal eosinophil positive rates without asthma symptoms did not increase by age raised.
• Nasal eosinophil positive have significant relationship with specific IgE positive and other allergic disease positive. It is easy to examine and low cost.
• Testing for nasal discharge eosinophils is easy and inexpensive, and similar to other predictive factors, with higher positive rates in older age groups. Moreover, a correlation was observed between nasal discharge eosinophils and other allergic disease markers (RAST positivity and coexisting diseases).

## 1 Data

### 1.1 Patients (Table 1.)

This data article included patients treated at Murayama Pediatrics with recurrent episodes of symptoms respiratory symptoms such as coughing, wheezing and dyspnea at least 3 recurrent episodes. And more than three times improvement using bronchodilator inhalation at intervals of ≥ 1 week. According to the Japanese guidelines for child asthma [1], which attaches importance for the early intervention of infant or child asthma, these patients were divided into an asthma group (A group) and a non-asthma group (NA group). A total of 180 patients showed nasal symptoms, such as nasal discharge for ≥ 1 week, without fever, being in 85 patients in the A group and 95 patients in the NA group.

### 1.2 Nasal eosinophil examination (Table 2)

Distribution of nasal discharge eosinophil counts in both data groups. The nasal discharge eosinophil counts in the entire cohort were: negative (−) in 60% of patients; +− in 12.9%; 1+ in 20.0%; 2+ in 2.3%; and 3+ in 4.7%. Most of the positive data were graded as either +− or +.

## 2 Experimental design, materials and methods

### 2.1 Experimental design and statistical analysis

Nasal eosinophil examination is routine test of allergic rhinitis, continued symptoms of nasal discharge without fever up. The data were pooled at Murayama Pediatrics from August to December 2008. This data were approved by the staff of Maruyama Pediatrics. Informed consent was received orally or in written form from all patients and/or their guardians. Using these data we investigated that nasal eosinophil can be a prognosis factor as well as allergic family history, coexisting other allergic diseases, IgEs and eosinophil in peripheral blood.

Statistical analysis using the chi-square test was performed with JSTAT for Windows version 17.1.

### 2.2 Material and methods

To examine that nasal eosinophil is a prognosis factor for persistent asthma, the pooled data were divided two groups according to with or without asthma symptoms by each age. We investigated that nasal eosinophil positive rates in asthma and non-asthma group by each age. Moreover we compared relationship of nasal eosinophil positive rates pattern with other prognosis factors.

### 2.3 With (A group) and without (NA group) asthma symptoms nasal eosinophil positive rates by each age (Fig. 1.)

The positive nasal discharge eosinophil rates according to age in the A group were: age 0–1 years, 14.3% (4/28); age 2–3 years, 47.8% (11/23); age 4–5 years, 42.1% (8/19) and age ≥ 6 years, 73.3% (11/15). The positive nasal discharge eosinophil rates increased with increasing age, the positive nasal discharge eosinophil rates in the A group were significantly higher for patients aged 2–3 years, 4–5 years, and ≥ 6 years than in patients aged 0–1 years (*p*< 0.05–0.001, chi-square test). Meanwhile, the positive nasal discharge eosinophil rates according to age in the NA group were approximately the same across all age groups (approximately 30% in each age group).

### 2.4 With (A group) and without (NA group) asthma symptoms IgE RAST positive rates by each age (Fig. 2.)

The RAST-positive rates, according to age, are shown in Fig. 2. The RAST reactions were positive (> 0.35 UA) mainly for egg whites, *Dermatophagoides farinae*, and cat skin scraps. The positive RAST rates according to age in the A group were age 0–1 years 21.4% (6/28), age 2–3 years 43.5% (10/23), age 4–5 years 42.1% (8/19) and age ≥ 6 years 93.3% (14/15). These positive RAST rates was significantly higher in the age ≥ 6 years groups than in the 0–1-year-old age group (*p*< 0.001). The positive age-based RAST rates in the NA group were approximately 0–10% overall and did not differ significantly between each age group. According to each age group, the RAST-positive rates on A group were significantly higher than those of NA group (*p*< 0.05–0.001).

**Fig. 1:**
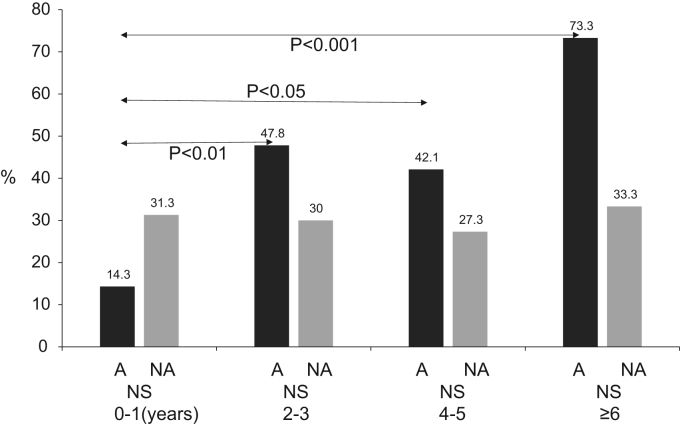
Nasal discharge eosinophils by age. A, Asthma group; NA, Non-asthma group. The positive nasal discharge eosinophil rates in the A group were significantly higher in patients aged 2–3 years, 4–5 years, and ≥ 6 years than in patients aged 0–1 years (*p*< 0.05-0.001, chi-square test).

**Fig. 2:**
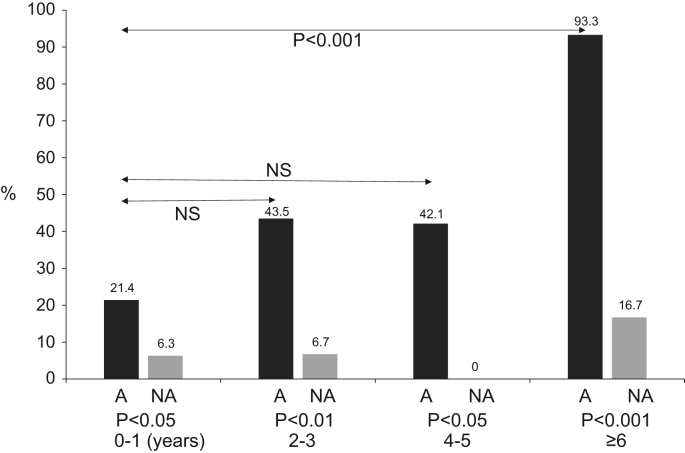
RAST-positive rates by age. A, Asthma group; NA, Non-asthma group. Positive RAST rates were significantly higher in the older age groups than in the 0–1-year-old age group (NS, *p*< 0.01).

### 2.5 With (A group) and without (NA group) asthma symptoms family history rates by each age (Fig. 3.)

**Fig. 3:**
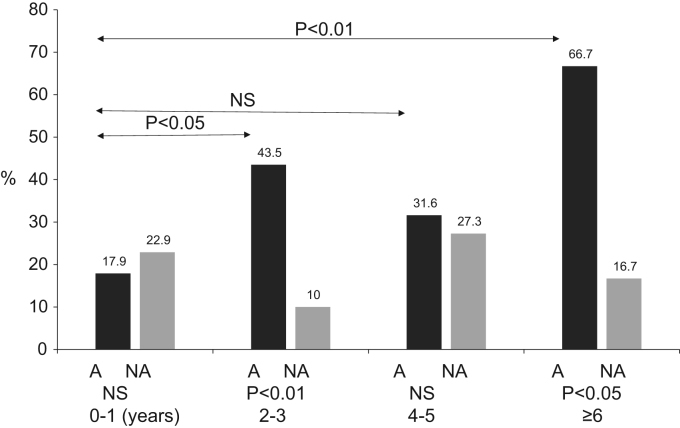
Positive allergic family history rates according to age group. A, Asthma group; NA, Non-asthma group. Positive allergic family history rates were significantly higher in the older age groups than in the 0–1-year-old age group (NS, *p*< 0.01).

The positive allergic family history rates, according to age, are shown in Fig. 3. The positive allergic family history rates, according to age, in the A group were age 0–1 years 17.9% (5/28), age 2–3 years 43.5% (10/23), age 4–5 years 31.6% (6/19) and age ≥ 6 years 66.7% (10/15). These positive allergic family history rates were significantly higher in the older age groups than in the 0–1-year-old age group (NS, *p*< 0.01). The positive allergic family history rates in the NA group were approximately 20% overall and did not differ significantly by age group. For each age group, the positive allergic family history rates of A group were higher than those of age matched NA group (NS, *p*< 0.01), except for the 0–1-years-old age group.

### 2.6 With (A group) and without (NA group) asthma symptoms coexisting other allergic diseases rates by each age (Fig. 4.)

**Fig. 4:**
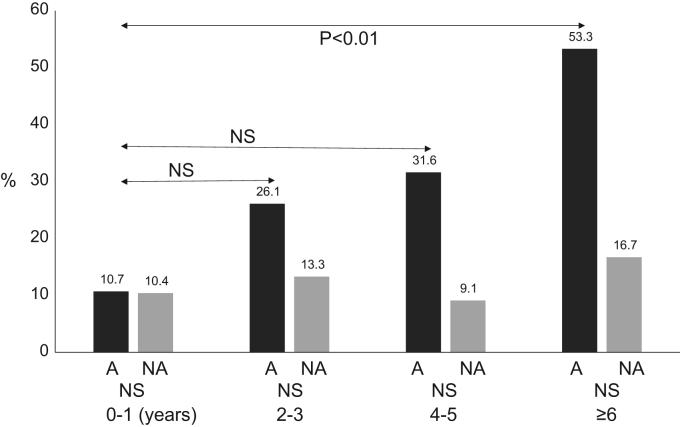
Positive coexisting or past allergic diseases rates (eczema, allergic conjunctivitis). A, asthma group; NA, Non-asthma group. The rates of coexisting allergic diseases were significantly higher in the older age groups than in the 0–1-year-old age group (NS, *p*< 0.01).

**Fig. 5:**
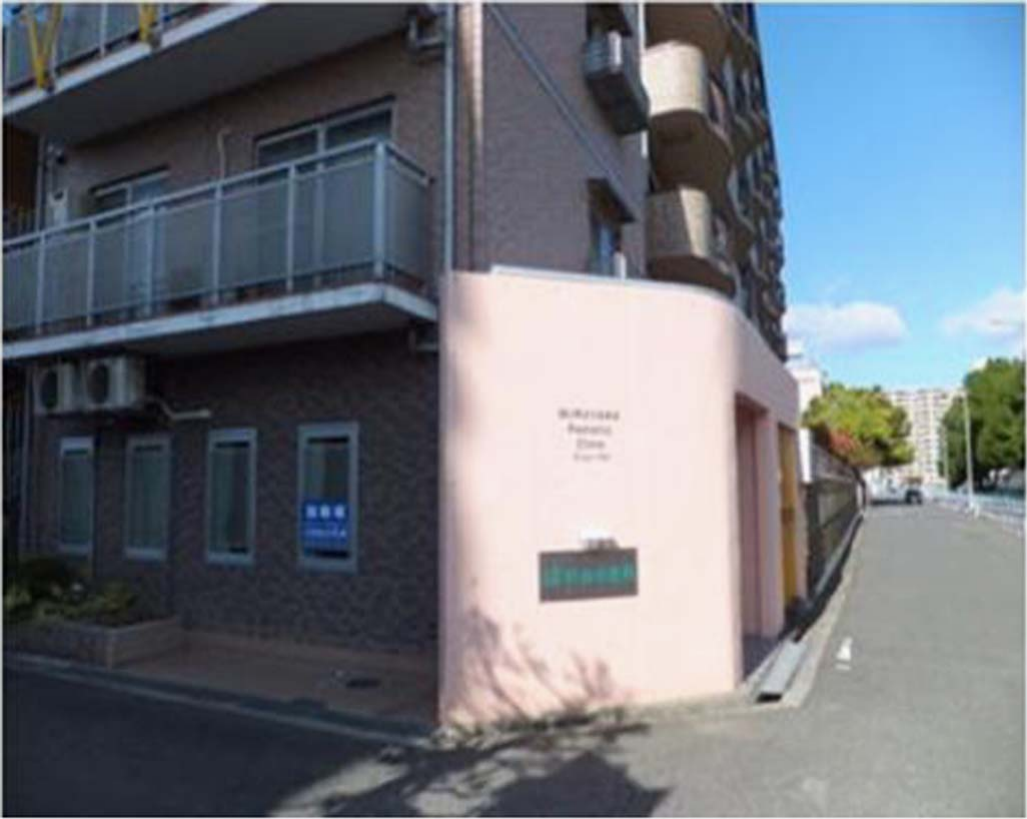
Murayama Pediatrics, Since 1994, Osaka, Japan. This is our Murayama Pediatric Clinic. In this clinic all nasal eosinophil data were gathered.

**Fig. 6:**
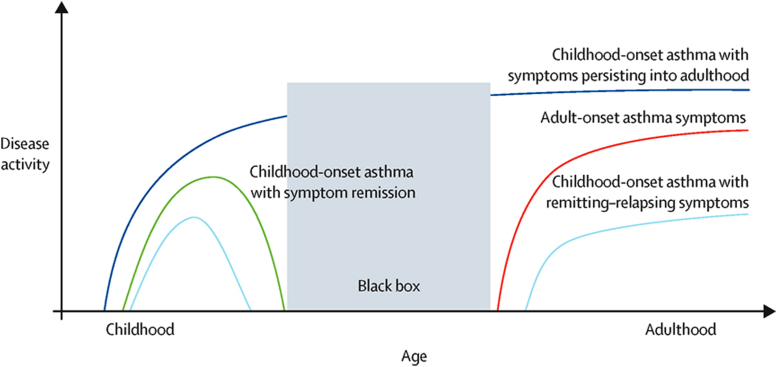
Prognosis of childhood asthma. Some infant asthma grow up to persistent asthma by age raised. The other infant asthma grow up to asthma symptoms free by age raised. Prognosis (predictive) factors play an important roles weather asthma symptoms are persistent or free.

The positive rates of coexisting or past allergic diseases (eczema, allergic conjunctivitis) are shown in Fig. 4. The positive coexisting allergic disease (other than asthma) rates in the A group were age 0–1 years 10.7% (3/28), age 2–3 years 26.1% (6/23), age 4–5 years 31.6% (6/19) and age ≥ 6 years 53.3% (8/15). The other coexisting allergic diseases rates were significantly higher in age ≥ 6 years, but not significant in any other older age group, when compared to the 0–1-year-old age group (*p*< 0.01) (Fig. 5, Fig. 6).

### 2.7 Correlation nasal eosinophil and other prospective factors in group A (Table 3.)

Each of the above 4 assessments in the A group showed significantly higher positive rates in each age group older than the 0–1-year-old age group. On the other hand, each of the above assessments in the NA group showed similar positive rates across all age groups, without significant differences. According to each age group, the positive rates of allergic family history rates on A group were not significantly different, when compared with those of the NA group.

Correlations between nasal discharge eosinophils and other predictive factors in the A group are presented in Table 3. In the A group, no significant correlation was observed between nasal discharge eosinophils and an allergic family history. However, significant correlations were observed between nasal discharge eosinophils and the RAST data (*p*< 0.01), as well as other coexisting or past allergic diseases (*p*< 0.05). In other words, positive nasal discharge eosinophil rates were significantly correlated with RAST positivity and the presence of coexisting or past allergic diseases.

**Table 1:** The number of patients in each age group.

Age (*y*)	0–1	2–3	4–5	> 6	Sum	Total
Asthma	28	23	19	15	85	180
No Asthma	48	30	11	6	95	(M: 94, F: 86)

**Table 2:** Nasal eosinophil counts for each group.

Eosinophil count	0	< 5/10F	5–50/10F	50–100/10F	>100/10F
By microscope (400×)		+-	+	++	+++
Asthma (%)	60.0	12.9	20.0	2.3	4.7
No Asthma (%)	69.4	16.8	11.6	1.1	1.1

An eosinophil count 0/10 F was considered negative, and eosinophil counts greater than 1/10 F were considered positive.

**Table 3:** Match correlations between nasal eosinophils and other predictive features.

A group	RAST Number of patients		Family Number of patients		Other Allergy Number of patients	
	Negative	Positive	Negative	Positive	Negative	Positive
Nasal eosinophil (+)	11	23**	18	16	20	14*
Nasal eosinophil (-)	36	15	36	15	42	9
	RAST Number of patients		Family Number of patients		Other Allergy Number of patients	
						
NA group	Negative	Positive	Negative	Positive	Negative	Positive
Nasal eosinophil (+)	27	2	23	6	24	5
Nasal eosinophil (-)	62	4	54	12	60	6

A, Asthma group; NA, Non-asthma group; Family, Atopic family history; Other, Other allergic diseases.

* *p*< 0.05,

** *p*< 0.01, Chi-squared test.
