# Supplementary material for: Data on nasal eosinophil positive rates in childhood asthma on each age
Source: Data Brief. 2018 Sep 13;20:1573–9. doi: 10.1016/j.dib.2018.09.018 (PMC6153190; doi:10.1016/j.dib.2018.09.018)
Supplement: Supplementary file 1 — Supplementary material [file mmc1.docx]

Conflict of interest

Title; Data on nasal eosinophil in young child asthma as a prognosis factor in old child asthma.

Author; Norihide Murayama MD

Institution; Murayama Pediatrics

3-2-33 Nagayoshi-Nagahara-Higashi Hirano-ku Osaka-shi Osaka 547-0013 Osaka Japan

E-mail; [norihide99@yahoo.co.jp](mailto:norihide99@yahoo.co.jp), TEL +81-6-6790-8835, Fax; +81-6-6790-8889

Authorship statement

This study was planned and conducted by Norihide Murayama and Kikuno Murayama.

Disclosure statement

I have no conflict of interest and I received no fund from anywhere about this study.

DiB follows the ICMJE recommendations regarding conflict of interest disclosures. All authors are required to report the following information with each submission:

1. All third-party financial support for the work in the submitted manuscript.

Answer; No

1. All financial relationships with any entities that could be viewed as relevant to the general area of the submitted manuscript.

Answer; No

1. All sources of revenue with relevance to the submitted work who made payments to you, or to your institution on your behalf, in the 36 months prior to submission.

Answer; No

1. Any other interactions with the sponsor of outside of the submitted work should also be reported.

Answer; No

1. Any relevant patents or copyrights (planned, pending, or issued).

Answer; No

1. Any other relationships or affiliations that may be perceived by readers to have influenced, or give the appearance of potentially influencing, what you wrote in the submitted work.

Answer; No

As a general guideline, it is usually better to disclose a relationship than not. This information will be acknowledged at publication in a Transparency Document link directly in the article

16 Aug 2018

Norihide Murayama MD
